# Pharmaceutical Co-crystal of Ketoconazole-adipic Acid: Excipient Compatibility and *In Silico* Antifungal Potential Studies

**DOI:** 10.1007/s11095-025-03910-7

**Published:** 2025-09-04

**Authors:** Flavia Martin, Maria Miclaus, Ana Maria Raluca Gherman, Monica Dan, Ioana Grosu, Xenia Filip, Irina Kacso

**Affiliations:** National Institute for Research and Development of Isotopic and Molecular Technologies, 67-103 Donat, 400293 Cluj-Napoca, Romania

**Keywords:** DSC, excipient compatibility, FTIR spectroscopy, ketoconazole-adipic acid co-crystal, molecular docking, powder x-ray diffraction, stability

## Abstract

**Objective:**

This research aimed to investigate the compatibility of the Ketoconazole-Adipic Acid (KTZ-AA) co-crystal, which exhibits an improved dissolution profile over pure Ketoconazole, with various solid pharmaceutical excipients, as well as its *in silico* antifungal potential.

**Methods:**

Binary physical mixtures (1:1 w/w) of KTZ-AA co-crystal and excipients were analyzed using differential scanning calorimetry (DSC), thermogravimetric analysis (TGA), Fourier-transform infrared spectroscopy (FT-IR), and powder X-ray diffraction (PXRD). The molecular docking study targeting the sterol 14α-demethylase (CYP51) enzyme of the pathogenic yeast *Candida albicans* was performed.

**Results:**

DSC results indicated compatibility between co-crystal and six tested excipients: lactose monohydrate, polyvinylpyrrolidone K90, microcrystalline cellulose, corn starch, colloidal silicon dioxide, and talc. In the case of the co-crystal and magnesium stearate mixture, DSC revealed a change in the thermal behavior, suggesting the formation of a eutectic system. However, TGA demonstrated that the decomposition profile of the co-crystal remained unaffected in all binary mixtures. PXRD and FT-IR further confirmed the absence of chemical interactions between the co-crystal and all excipients under ambient conditions. Moreover, the KTZ-AA co-crystal maintained its chemical stability without degradation after three months storage under accelerated conditions (40°C/75% RH). The molecular docking study demonstrated that co-crystallization of KTZ with AA enhances its binding affinity to CYP51 enzyme compared to KTZ alone.

**Conclusion:**

The excipient compatibility study conducted on the Ketoconazole-Adipic Acid co-crystal confirmed its potential for development as a solid oral dosage form with improved antifungal activity, presenting a promising alternative to the parent drug.

**Supplementary Information:**

The online version contains supplementary material available at 10.1007/s11095-025-03910-7.

## Introduction

The development of a successful pharmaceutical product is reflected by its efficacy, safety, quality and stability and involves a detailed characterization of the physicochemical properties for the active drug, *i.e.* Active Pharmaceutical Ingredient (API) together with all constituents in direct contact with it, including additives, excipients, or packaging substances [1]. Over 80% of commercially pharmaceutical drugs are produced as solid formulations intended for oral administration due to their high-precision dosing, versatility, fabricating efficiency and most importantly, patient compliance [2].

To ensure effective therapy, any API must possess adequate solubility and permeability to be produced as solid pharmaceutical dosage form in order to provide suitable bioavailability in the systemic circulation [3]. Based on Biopharmaceutical Classification System (BCS), APIs having solubility/permeability issues fall into Class II and Class IV [4], therefor their bioavailability is of utmost importance to be improved but without altering the chemical identity and pharmacokinetic properties of the native drug molecule. In this context, crystal engineering represents a viable technique in pharmaceutical research aimed to enhance the solubility and other physicochemical properties of APIs [5, 6] by multicomponent drug products formation, *e.g*., polymorphs, hydrates/solvates [7, 8], salts [9, 10], co-crystals [11–14], or co-amorphous solid dispersions [15, 16].

The API-excipient compatibility screening constitutes a mandatory part of preformulation phase for any new drug manufacturing process aimed to provide a stable final product [17, 18]. The principles guiding formulation development were outlined and established according to ICH Q8 (R2) guidelines [19, 20]. Excipients serve various purposes and are incorporated into pharmaceutical dosage forms in larger amounts compared to API, ranging from 1 to 99 percent by weigh [21]. Although generally claimed as pharmacologically inert, these components can be involved in physical and/or chemical interactions with the API, thus negatively affecting the stability or therapeutic performance of the active drug [1, 22].

The study of drug-excipient interactions (DEIs) involves combining the therapeutic agent with an excipient as a physical mixture, in a defined ratio, followed by solid samples exposure to various stress conditions, such as elevated temperature and humidity, thus activating the potential DEI or associated incompatibilities [23, 24]. Some studies revealed that adsorbed moisture can interact with the pure API altering its properties while high temperature may conducts to faster degradation [25–27]. Physical type interaction may impact the uniformity of the processed dosage form and influence critical parameters like the dissolution rate, ultimately affecting the drug’s bioavailability [28, 29]. The chemical type of interaction can generate API degradation while harmful impurities are produced, or possible reactions between ionizable APIs and ionized excipients can occur, leading to the generation of insoluble products [30]. The physicochemical and performance characteristics of these drug-excipient physical mixtures are evaluated combining various thermo-analytical and spectroscopic techniques, *e.g.,* differential scanning calorimetry (DSC), thermogravimetric analysis (TGA), isothermal microcalorimetry, Fourier transform infrared spectroscopy (FT-IR), solid state NMR, powder X-ray diffraction (PXRD), before and after equilibration [31, 32]. Therefore, the careful selection of excipients is thus mandatory to avoid the negative effects which can arise from drug-excipient incompatibility, and to maximize the chance for developing an optimal pharmaceutical formulation.

In the context of improving APIs solubility by crystal engineering approach, we explored the propensity for salt/co-crystal formation of the imidazole-type antifungal agent, namely *Ketoconazole* (KTZ). Introduced by FDA in 1981, KTZ was the first azole orally available for treating systemic fungal infections. It acts by inhibiting the ergosterol biosynthesis, a critical constituent of fungal cell membranes [33]. According to BCS is classified as a class II drug, with high permeability but low aqueous solubility (0.017 mg/mL [34]), since its dissolution properties in the gastrointestinal tract are insufficient under normal conditions [4, 35]. All this are reflected in its low bioavailability, and in addition, the hepatotoxicity of KTZ required a reconsideration of its commercial oral formulations. However, the KTZ benefits outweigh its side effects, leading to its off-label use as a second-line therapy for castration-resistant prostate cancer [36], and in 2014 the European Medicines Agency approved KTZ HRA for oral use to treat endogenous Cushing’s syndrome [37].

To date, we have reported several crystalline solid forms of KTZ with pharmaceutically relevant or generally recognized as safe (GRAS) coformers. These include a salt with oxalic acid, and co-crystals with dicarboxylic acids [38] and *p*-aminobenzoic acid (PABA), [39], as well as a supramolecular complex based on PAMAM dendrimer [40]. Among these developed formulations, two co-crystals, formed with adipic acid and fumaric acid, exhibited a 100-fold aqueous solubility improvement over pure KTZ [38]. Moreover, the KTZ-PABA co-crystal demonstrated its potential to be use as an alternative antifungal topical agent *versus* the commercial formulation [41]. Therefore, increasing the solubility and, obviously, the bioavailability of KTZ could decrease the required therapeutic dose and, at the same time, provide hepatoprotection.

**Fig. 1:**
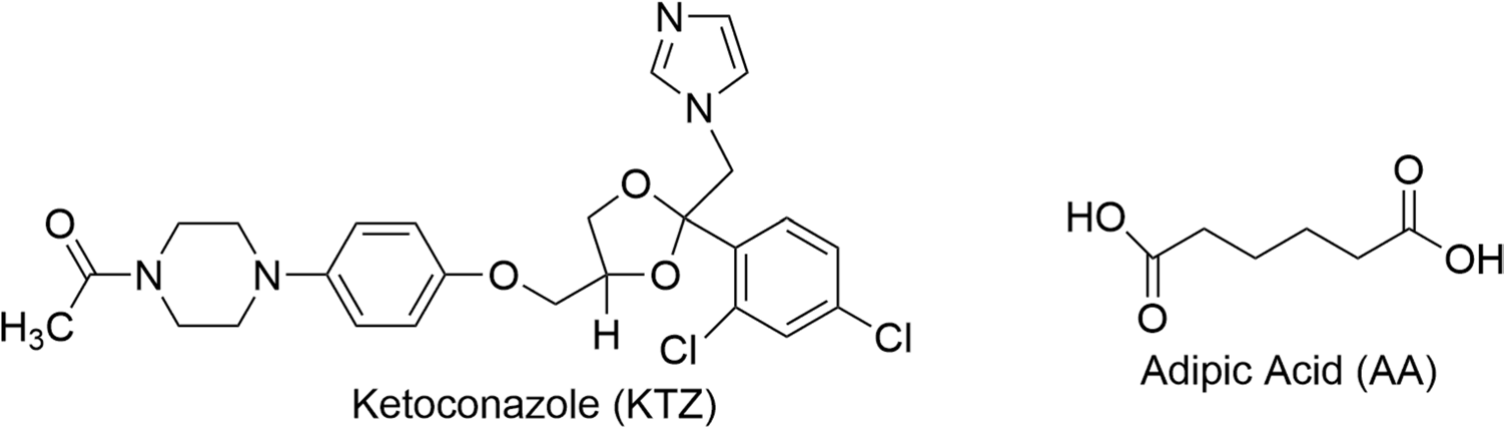
Molecular structures of Ketoconazole and Adipic Acid coformer.

. Based on our previously reported promising results, this study aims to develop a robust solid oral dosage form of the KTZ-Adipic Acid (KTZ-AA) co-crystal [38] (Fig. 1). We proposed to investigate its compatibility with several pharmaceutical excipients, particularly those used in commercial oral formulations of KTZ, along with molecular docking study to predict the co-crystal’s binding affinity and binding energy toward biological molecules.

As in case of pure crystalline APIs, the study of DEI is also mandatory for salts or co-crystals forms when such dosage formulations are promoted as potential pharmaceutical products for market approval [42–44]. A review of the literature reveals an increased interest in co-crystal-excipient compatibility testing [24, 45–47]. At present, at least ten formulations based on pharmaceutical co-crystals have been approved by the FDA and are commercially available, and more promising are in clinical trials [12, 14, 44].

The compatibility study of the KTZ-AA co-crystal was investigated with seven selected excipients, having various functions in the development of solid formulations, namely magnesium stearate (MgSt), lactose monohydrate (Lactose), polyvinyl-pyrrolidone K90 (PVP K90), microcrystalline cellulose (MCC), corn starch (Starch), colloidal silicon dioxide (SiO_2_), and Talc. These selected excipients are particularly relevant, as they are used in commercial oral formulations of KTZ.An initial rapid assessment of the interactions between KTZ-AA and the selected excipients was performed using the thermal methods DSC/TGA, followed by analytical ones, such as PXRD and FTIR. The stability of these binary physical mixtures were further tested under both ambient and accelerated storage conditions (temperature/relative humidity 40°C/75% RH). Additionally, after the storage period, the moisture content of each formulated mixture was determined by Karl Fischer (KF) titration [48]. The thermo-analytical and spectroscopic techniques used in this study offer the advantage of rapid analysis and require small sample sizes.

## Materials and Methods

### Materials

Ketoconazole, active drug, was obtained from Melone Pharmaceutical Co. Ltd., China. Adipic Acid coformer and colloidal silicon dioxide excipient from Merck KGaA, Germany. The other excipients, magnesium stearate, lactose monohydrate, polyvinyl-pyrrolidone K90, microcrystalline cellulose, corn starch, and talc were purchased from TCI Co.Ltd, Japan. All the compounds were used as received, without further purification. The solid excipients were milled and sieved through a 0.2 mm mesh prior to use.

### Co-crystal-excipient Solid Mixtures Preparation

Co-crystal-excipients compatibility studies were conducted on binary physical mixtures prepared by combining pure KTZ-AA with the selected excipients: MgSt, Lactose, PVP K90, MCC, Starch, SiO_2_, and Talc in a 1:1 (w:w) ratio. Equivalent amounts of each component were gently homogenized by dry grinding in an agate mortar with pestle for 5 min at room temperature, then stored in amber glass vials. This selected 1:1 ratio is usually chosen in order to maximize the occurrence of potential interactions [49, 50].

### Compatibility Study Methods

Thermal behavior was analyzed with a DSC-60 Shimadzu differential scanning calorimeter and a SDT Q600 TA Instruments thermogravimeter. For the DSC measurements an amount about 1.5–1.7 mg of each sample and the reference material (alumina) were placed in standard aluminum crimped pans and were heated with 10°C min^−1^ rate in the 20–300°C temperature range under dry nitrogen flow (3.5 L h^−1^). Data collection and analysis were performed using Shimadzu TA-WS60 and TA60 2.1 software. Thermogravimetric analysis in the 30–600°C temperature range with 10°C min^−1^ heating rate was performed in air (with 12% oxygen) flow of 20 mL min^−1^, using an amount of 8.0 ± 1.0 mg sample placed in alumina cell.

Rigaku SmartLab multipurpose diffractometer equipped with a 9 kW rotating anode was used for X-ray diffraction patterns collection, at room temperature, using Cu Kα1 radiation (λ = 1.54056 Å). The each ground sample as a fine homogeneous powder was mounted on a sample holder and was measured in the 3°–40° 2θ range with 0.01°.steps. For acquisition of the experimental data the Smart- Lab Guidance software was employed.

Spectroscopic characterization in the 4000–400 cm^−1^ spectral domain was performed with a JASCO 6100 FT-IR spectrometer with a resolution of 4 cm^−1^ using the KBr pellet technique. Pellets were obtained by dispersing 1 mg amount of each sample in about 300 mg of anhydrous KBr, grinding the mixtures in an agate mortar, followed by pressing in an evacuated die. The IR spectra were collected and analyzed using Jasco Spectra Manager v.2 software.

The stability testing was carried out in two ways: under ambient conditions by keeping the samples at room temperature for 6 months, and under accelerated conditions by storing the samples at elevated temperature and humidity (40°C and 75% RH) for 3 months in a Memmert HCP10 climate chamber.

Water content determination by Karl Fischer coulometric titration method has been performed with TitroLine 7500 KF trace apparatus using iodine, sulfur dioxide and pyridine in methanol.

The X-ray diffraction patterns were used for determination of the degree of crystallinity of the binary mixtures based on background subtraction method using the Powder Crystallinity module implemented in the Material Studio software [51].

### Molecular Docking Study Methods

The protein structure of sterol 14α-demethylase (CYP51) from the pathogenic yeast *Candida albicans*[PDB id: 5FSA] [52] was used as the receptor and was sourced from the Protein Data Bank. All water molecules, the original ligand, and other cofactors were omitted from the original file. KTZ, AA, and KTZ-AA co-crystal were selected as ligands, their structures being those previously obtained by our group [38].

Posaconazole, the original complexed antifungal drug, was docked as well in the validation of the binding site step. All ligand geometries were optimized in gas phase by using B3LYP hybrid exchange–correlation functional [53–56] paired with the 6-31G(d,p) basis set, as implemented in Gaussian software 16 Rev. C.01/C.02 [57].

Autodock Tools 4 [58] was used for building the ligand-receptor systems, adding only polar hydrogens to the receptor structure. The receptor was kept rigid and added Kollman charges to it. In contrast, the ligands were given complete flexibility: 13 torsion angles were set for posaconazole, 7 for AA, 8 for KTZ, 17 for KTZ-AA co-crystal.

The active site identified by the original inhibitor (posaconazole) in the crystallized complex was initially confirmed to ensure accurate binding of the studied ligands to sterol 14-α demethylase. Firstly, posaconazole was removed from the crystallized ligand-receptor complex, followed by a molecular docking scan within a search box that included the entire receptor. The overlaid redocked original ligand over the crystallized complex confirmed the binding site position. The final search box comprising the validated binding site was set up at coordinates (x,y,z) = (193,3,38) having the size of 26 × 26x26 Å, with a grid spacing of 0.375 Å (standard value). Autodock Vina algorithm [59] was used to dock KTZ, AA, and co-crystal to sterol 14-α demethylase.

## Results and Discussion

### Co-crystal—excipient Compatibility Study

The preparation method used in this study involved gently mixing the components in an agate mortar with pestle under ambient conditions. Although it is known that the polymorphic form of an API can potentially change [25, 60], this possibility was addressed by initially preparing a KTZ-AA sample through gentle mixing of the dry co-crystal powder in a mortar with pestle for 5 min at room temperature. DSC, PXRD and FTIR techniques confirmed that the polymorphic form of the KTZ-AA co-crystal remain unchanged.

### Differential Scanning Calorimetry

DSC is usually employed as a first technique for rapidly screening of interactions/incompatibilities between the components of an API-excipient binary physical mixture, as well as for assessing the thermal stability of the mixture. The incompatibilities are highlighted by changing of the thermal characteristics, such as appearance, disappearance or shifting of DSC peaks, or changes of the enthalpy of transition value, Δ*H* [61–63].

Table I presents the thermal events observed for KTZ-AA, the tested excipients and their binary physical mixtures through controlled heating.

**Table I:** Temperatures and Δ*H* vAlues of Melting Peak for KTZ-AA and Physical Mixtures in DSC Curve

Sample	*T*_onset_/ºC	*T*_peak_/ºC	Δ*H*/J g^−1^
KTZ AA KTZ-AA	147.5 151.6 127.0	149.5 153.0 128.6	− 115.9 − 360.3 − 118.07
MgSt	52.5	66.3	− 84.46
KTZ-AA—MgSt	90.7 111.4	105.3 120.1 129.7	− 35.90 − 10.58
Lactose	142.9 163.6 170.6 208.6	146.6 173.1 176.7 215.5	− 121.6 − 28.7 15.44 − 137.9
KTZ-AA—Lactose	125.0 127.5	142.9 144.9	− 45.11 − 50.73
PVP K90	40.0	56.6	− 111.87
KTZ-AA—PVP K90	33.8 121.7	59.3 127.1	− 70.96 − 40.39
MCC	32.2	52.2	− 109.92
KTZ-AA—MCC	126.0	128.1	− 54.33
Starch	35.0	72.0	− 185.88
KTZ-AA—Starch	124.4	126.8	− 46.12
SiO_2_	45.0	56.6	− 29.74
KTZ-AA—SiO_2_	126.2	129.1	− 86.67
Talc	205.0	212.1	− 2.93
KTZ-AA—Talc	125.9	128.3	− 48.91

**Fig. 2:**
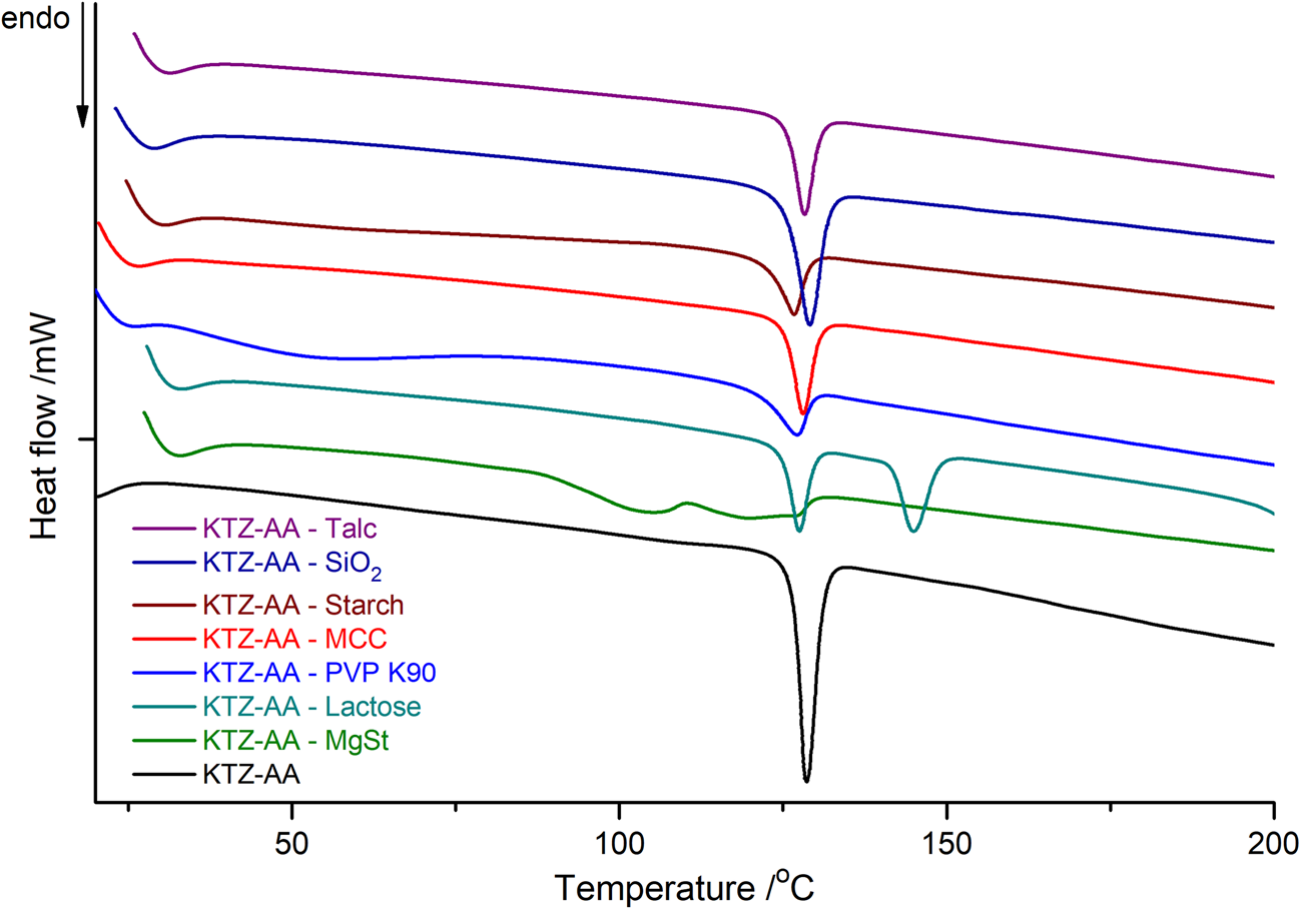
DSC curves of the KTZ-AA co-crystal and binary mixtures of KTZ-AA with tested excipients.

The DSC curve of the pure KTZ-AA co-crystal under controlled heating shows a sharp endothermic peak with *T*_on_ = 127.0°C and enthalpy of fusion Δ*H* = 118.07 J g^−1^ (Fig. 2), indicating thermal stability up to 200°C.

DSC analysis of the KTZ-AA co-crystal in binary mixtures with Lactose, PVP K90, MCC, Starch, SiO_2_ or Talc indicated no evidence of interactions between the components. The thermal characteristics remained practically unchanged, and the minor shifts in DSC peak position observed are typical for physical mixtures [24, 63].

**Fig. 3:**
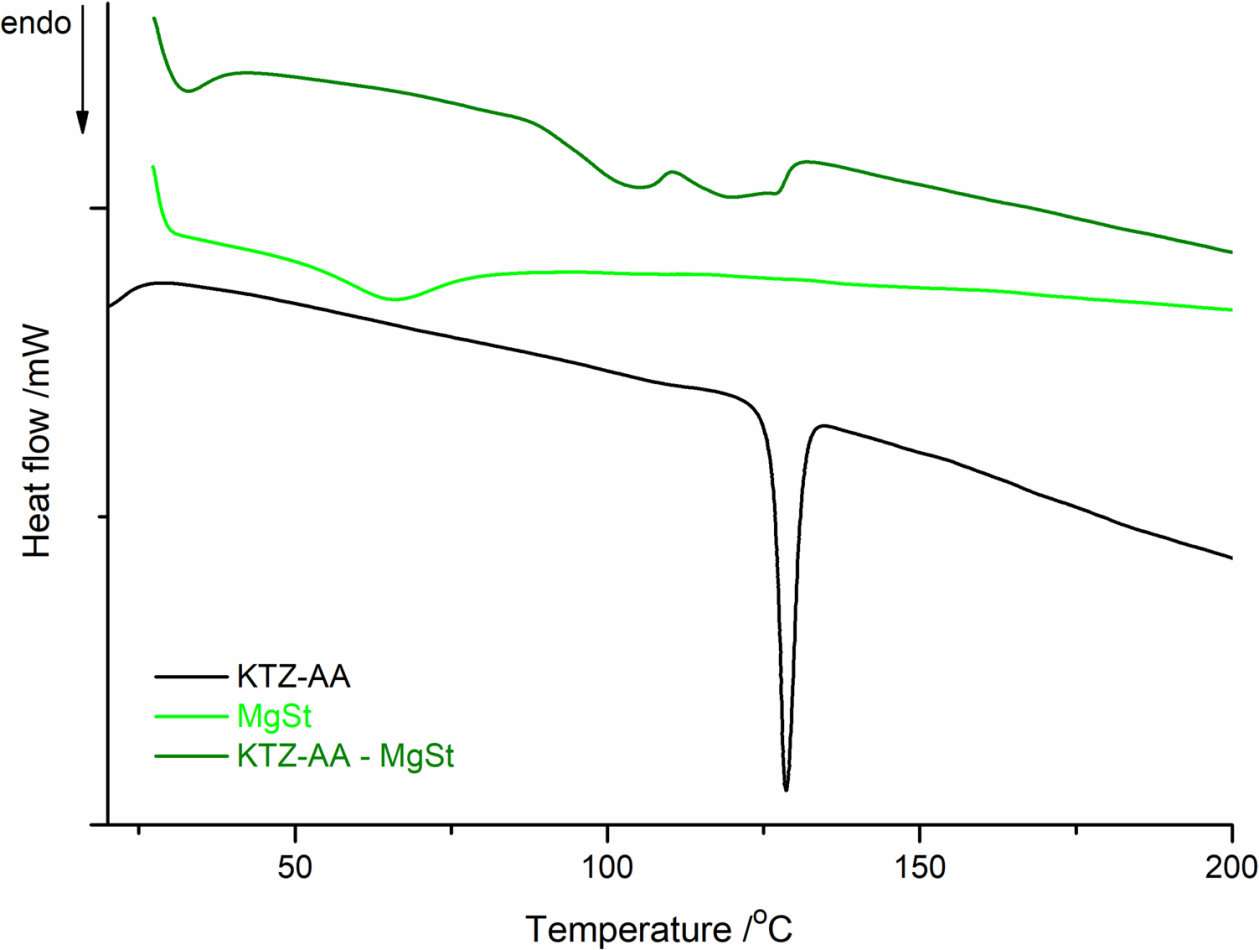
DSC curves of the KTZ-AA co-crystal, MgSt and KTZ-AA—MgSt binary mixture.

In the case of the KTZ-AA – MgSt binary mixture, the DSC trace showed a completely different thermal behavior relative to the pure components (Fig. 3). The DSC curve of pure MgSt exhibits a broad endothermic peak in the 50–80°C temperature range, with *T*_on_ = 52.5°C, peak maximum at 66.3°C and Δ*H* = 84.46 J g^−1^, corresponding to water loss from the sample. In contrast, the DSC curve of the binary mixture exhibits three broad endothermic events: the first between 87–110°C with *T*_peak_ = 105.3°C and Δ*H* = 35.90 J g^−1^, the second between 110 and 125°C with *T*_peak_ = 120.1°C and Δ*H* = –10.58 J g^−1^, and the third appearing as a shoulder with maximum at 129.7°C. Above 200°C, the sample decomposition occurred.

The disappearance of the co-crystal melting peak and the appearance of three new endothermic events for the KTZ-AA – MgSt mixture in the 85–130°C temperature range suggest some solid state interactions between the mixture components. This thermal profile is most likely attributable to the formation of a eutectic system involving the co-crystal and this excipient, and not due to a chemical interaction. Similar thermal behavior of the MgSt was encountered in other reported compatibility studies [23, 64]. These high-temperature interactions between KTZ-AA and MgSt observed are currently being further investigated using the other instrumental techniques (PXRD, FT-IR) under ambient conditions.

### Thermogravimetry

TGA technique was used to determine the thermal stability, decomposition behavior and the water content of the binary mixtures. The percentage mass loss of each formulation as a function of temperature is listed in Table II.

**Table II:** TGA Analysis Results of KTZ-AA and its Binary Mixtures with Excipients

Sample	Loss stage	Temperature range/ºC	Mass loss/%
KTZ-AA	1 2 3	140–260 260–390 390–660	15.40 38.37 46.11
KTZ-AA – MgSt	1 2 3 4 5 6 7	50–155 155–250 250–300 300–325 325–400 400–500 500–620	1.88 10.45 13.41 5.34 26.47 16.39 24.50
KTZ-AA – Lactose	1 2 3 4	105–150 150–260 260–380 380–660	2.58 33.38 16.94 46.14
KTZ-AA – PVP K90	1 2 3 4 5	30–70 80–300 300–375 375–470 470–665	6.07 8.23 16.25 40.70 28.17
KTZ-AA – MCC	1 2 3 4	30–75 170–255 255–350 350–670	1.99 8.27 39.19 48.91
KTZ-AA – Starch	1 2 3 4 5	30–90 160–245 245–335 335–375 375–660	5.57 6.09 35.27 9.69 41.36
KTZ-AA – SiO_2_	1 2 3	100–250 250–375 375–700	13.02 24.08 40.58
KTZ-AA – Talc	1 2 3	155–215 215–380 380–600	6.27 14.40 29.39

**Fig. 4:**
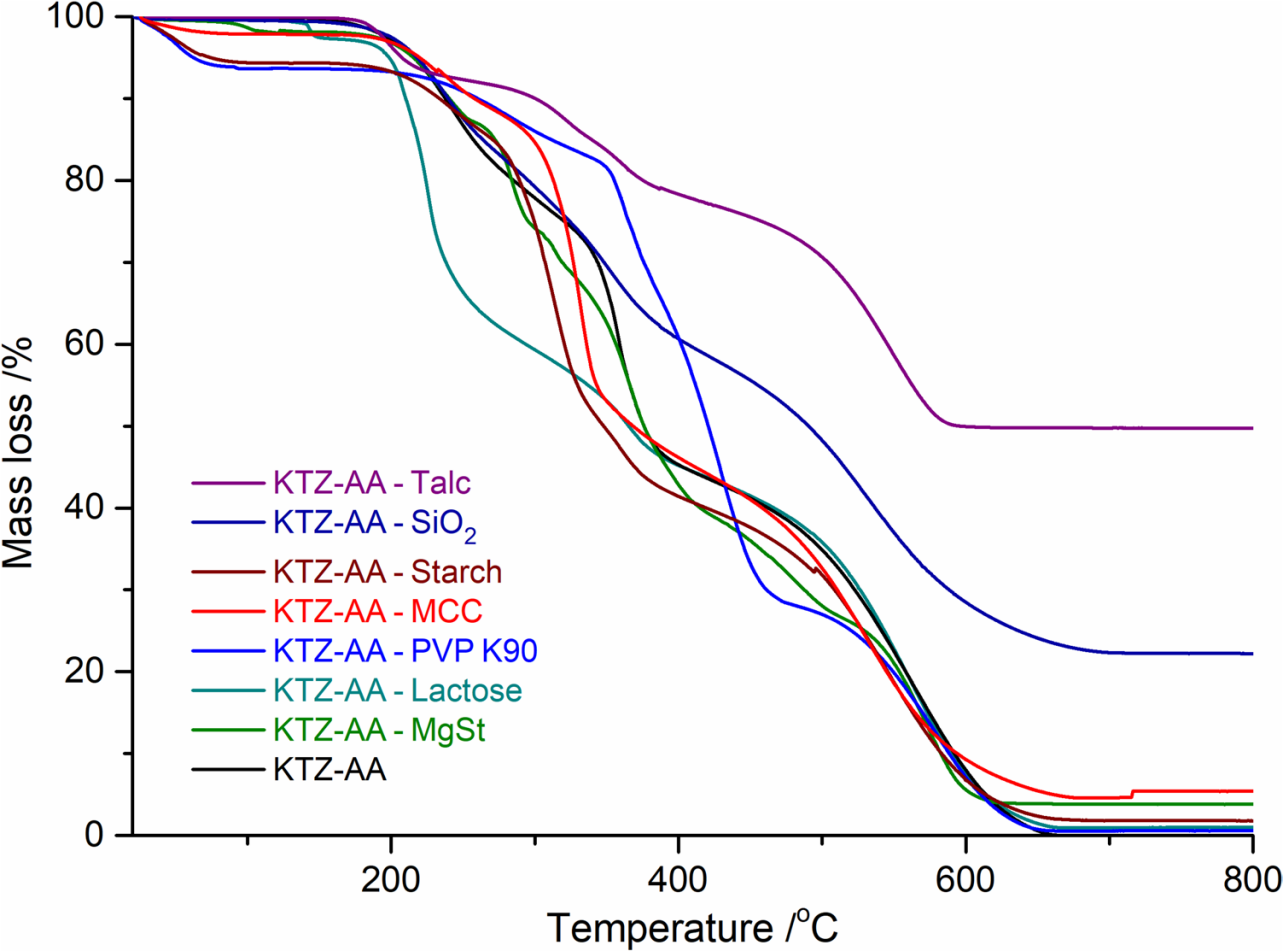
TGA curves of the physical mixture of KTZ-AA and excipients.

The recorded data indicate that the thermal stability of the KTZ-AA co-crystal remains unaffected when mixed with the tested excipients (Fig. 4), the thermal decomposition processes occurr both for the co-crystal and the binary mixtures above ~ 140°C. The initial mass loss observed at this temperature is due to the release of adsorbed water.

For all tested binary mixtures, the mass loss recorded up to 140°C, associated with water loss through drying, correlates well with the water content determined by the Karl Fischer method (see further Table III).

**Table III:** Water content (%) of physical mixtures before and after storage at accelerated stability conditions (at 40 °C/75% RH) determined by Karl-Fischer method and TGA analysis

Sample	Water content (%)		TGA mass loss up to 140 °C (%) before storage
	before	after	
	storage		
KTZ-AA - MgSt	2.2	4.3	1.875
KTZ-AA - Lactose	3.0	5.3	2.576
KTZ-AA - PVP K90	9.9	13.0	6.073
KTZ-AA - MCC	3.2	4.8	1.996
KTZ-AA - Starch	6.1	9.9	5.571
KTZ-AA - SiO_2_	0.9	2.1	0.000
KTZ-AA - Talc	0.9	2.1	0.000

### Powder X‑ray Diffraction

PXRD analytical technique is usually applied in the preformulation stage to characterize the crystalline nature of compounds, and at the same time, for APIs polymorph screening. Each crystalline substance presents a unique X-ray diffraction pattern, displayed by peak intensities against a range of diffraction angles (2*θ*). API-excipient interactions may conduct to changes in the API’s crystalline form, which appears as shifts, appearances or disappearances of the characteristic peaks. In order to determine any drug-excipient interactions that might occur, the PXRD pattern of the analyzed binary mixture is compared with those of the individual pure components.

**Fig. 5:**
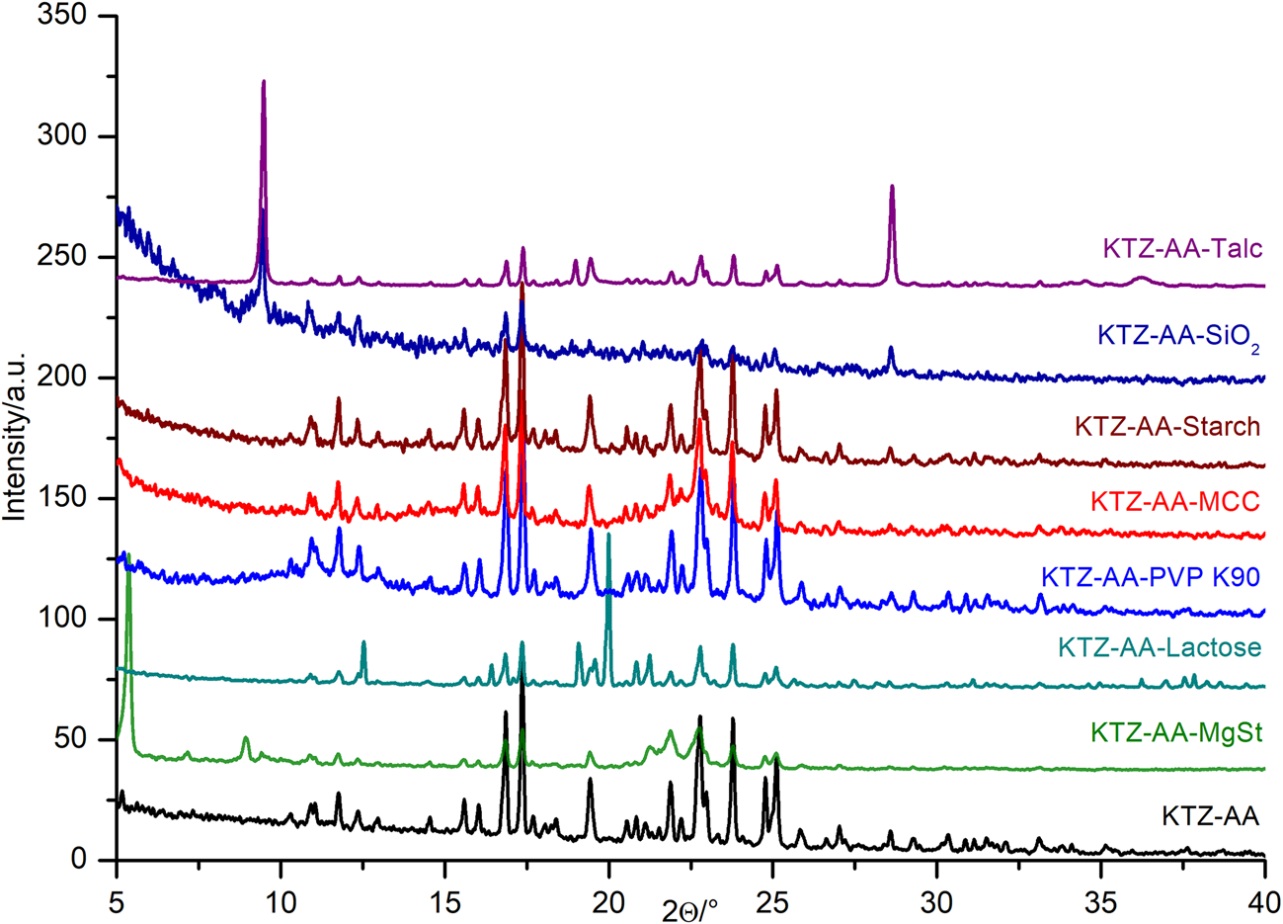
PXRD patterns of the KTZ-AA co-crystal and KTZ-AA—excipients physical mixtures.

The characteristic diffraction peaks of the KTZ-AA can be distinguished in all PXRD patterns of the binary mixtures, indicating no structural changes in the co-crystal caused by interactions with the excipients at room temperature. However, the PXRD patterns of the binary mixtures with MCC and PVP K90 show reduced crystallinity, due to the amorphous nature of these excipients. On the other hand, the mixture with MgSt exhibits broadening of the diffraction peaks (Fig. 5).

### FT‑IR Spectroscopy

FT-IR spectroscopy is a commomly used complementary technique for screening, drug-excipient compatibility. Changes observed in FT-IR spectra may be generated from drug-excipient chemical interactions, polymorphic transitions, hydrates/solvates formation, dehydration, or other processes [65, 66]. For compatible API-excipient binary mixtures, the FT-IR spectra typically represent a simple superposition of individual component spectra without anydisappearance, shift, or broadening of characteristic vibrational bands.

**Fig. 6:**
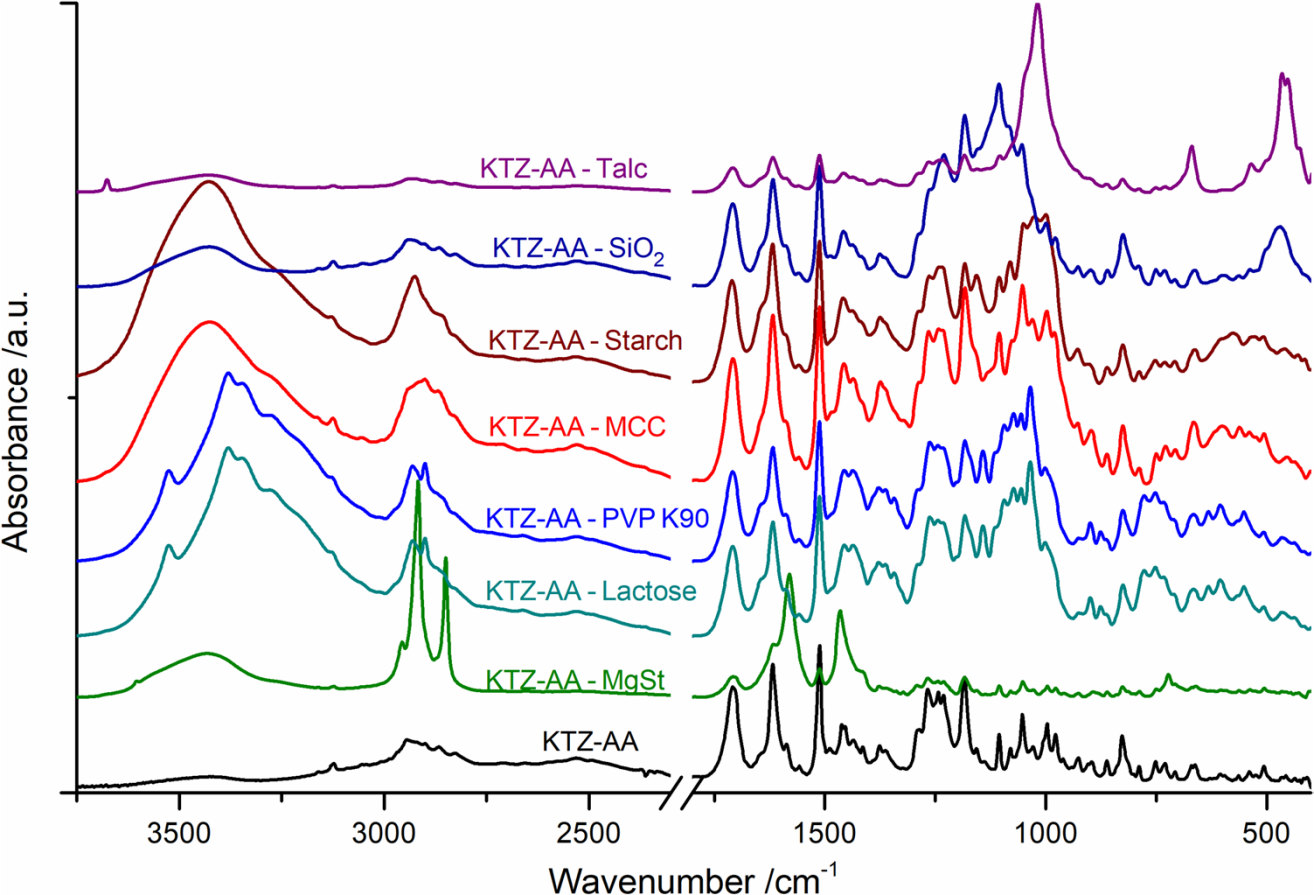
FT-IR spectra of the KTZ-AA co-crystal and binary mixtures of KTZ-AA with tested excipients.

The FT-IR spectra recorded for the pure KTZ-AA and the corresponding binary mixtures with the studied excipients are shown in Fig. 6.

The FT-IR spectra of all binary physical mixtures show only minor shifts of the characteristic vibrational bands assigned to pure KTZ-AA co-crystal and tested excipients, as listed in Table S1 [67–70]. These spectra essentially represent a superposition of the individual component spectra. Thus, FT-IR analysis further confirms the absence of chemical interactions between KTZ-AA and the studied excipients at room temperature.

### Stability, Water Content and Crystallinity

Stability is a mandatory requirement for any solid formulation intended for market release. The enhancements in solubility and bioavailability along with other significant advantages provided by a co-crystal form compared to the pure API are only guaranteed if the solid form is maintained during the production process, storage and commercialization prior to patient use [44].

A possible disadvantage of pharmaceutical co-crystals consist in their tendency to dissociate into their individual components, such as the free API and the corresponding conformer. Although co-crystals are generally stable in the solid state exposure to elevated temperature and high relative humidity (RH) can promote their dissociation [71–73]. The KTZ-AA co-crystal stability has been previously evaluated through storage under ambient conditions for 6 months and accelerated conditions (40°C/75% RH) for 4 months, according to European Medicines Agency Guidelines [74], as reported in our earlier study [38].

Considering that heat and moisture are the primary catalysts for drug-excipient interactions, the physical stability of the KTZ-AA – excipients mixtures was further investigated under two different storage conditions: (*i*) environmental (room temperature, exposure to intense light, and ambient humidity) for 6 months, and (*ii*) accelerated conditions (40°C and 75% RH) for 3 months. Following storage, the binary mixtures samples were analyzed by PXRD and no changes were observed in the position or intensity of the co-crystal peaks, indicating that no structural transformations occurred (Figure S1). These results confirm that the storage conditions do not affect the stability of the KTZ-AA—excipient binary mixtures.

The moisture content is a key factor which may affect the stability, solubility and overall the shelf life of pharmaceutical solid formulations. APIs sensitive to moisture can undergo hydrolysis or even form hydrates during manufacturing process, thus affecting the quality of the pharmaceutical formulation.

Karl Fischer titration is a frequently employed method for moisture content determination, based on the reaction between iodine and sulfur dioxide in presence of water [48]. In this study the water content was calculated for the co-crystal—excipient binary mixtures both before and after storage under accelerated conditions (climatic chamber, 40°C/75% RH, for 3 months). The initial moisture content values obtained by Karl Fischer titration were consistent with those determined by TGA analysis (Table III). Following storage under high humidity, the determined water content was mostly adsorbed moisture, present in the form of free water.

The degree of crystallinity was calculated using X-ray diffraction patterns data, applying the Background subtraction method. This method implements a simple, approximate algorithm to decompose the mixture pattern into three components: crystalline, amorphous and background scattering. The procedure involves two steps: (*i*) decomposition of the powder diffraction mixture pattern into these three coherent scattering contributions,; and (*ii*) calculation of the relative crystalline phase content using the separated components of the diffraction pattern of the mixture [51].

Following storage under accelerated conditions, the examined KTZ-AA—excipient mixtures presented a slightly decrease in crystallinity values compared to those measured before storage (Table 4). An exception was observed in the mixtures containing MgSt and Talc, which exhibited a slight increase in crystallinity values, probable due to the hygroscopic nature of these excipients.

**Table IV:** Degree of crystallinity of the KTZ-AA—Excipients Physical Mixtures before and after 3 Months Storage at Accelerated Stability Conditions (40°C/75% RH)

Sample	Degree of crystallinity/%	
	Before storage	After storage
KTZ-AA – MgSt	66.16	71.48
KTZ-AA – Lactose	81.27	70.13
KTZ-AA – PVP K90	70.15	68.58
KTZ-AA – MCC	69.54	58.67
KTZ-AA – Starch	71.23	61.51
KTZ-AA – SiO_2_	80.30	70.50
KTZ-AA – Talc	62.73	68.14

**Fig. 7:**
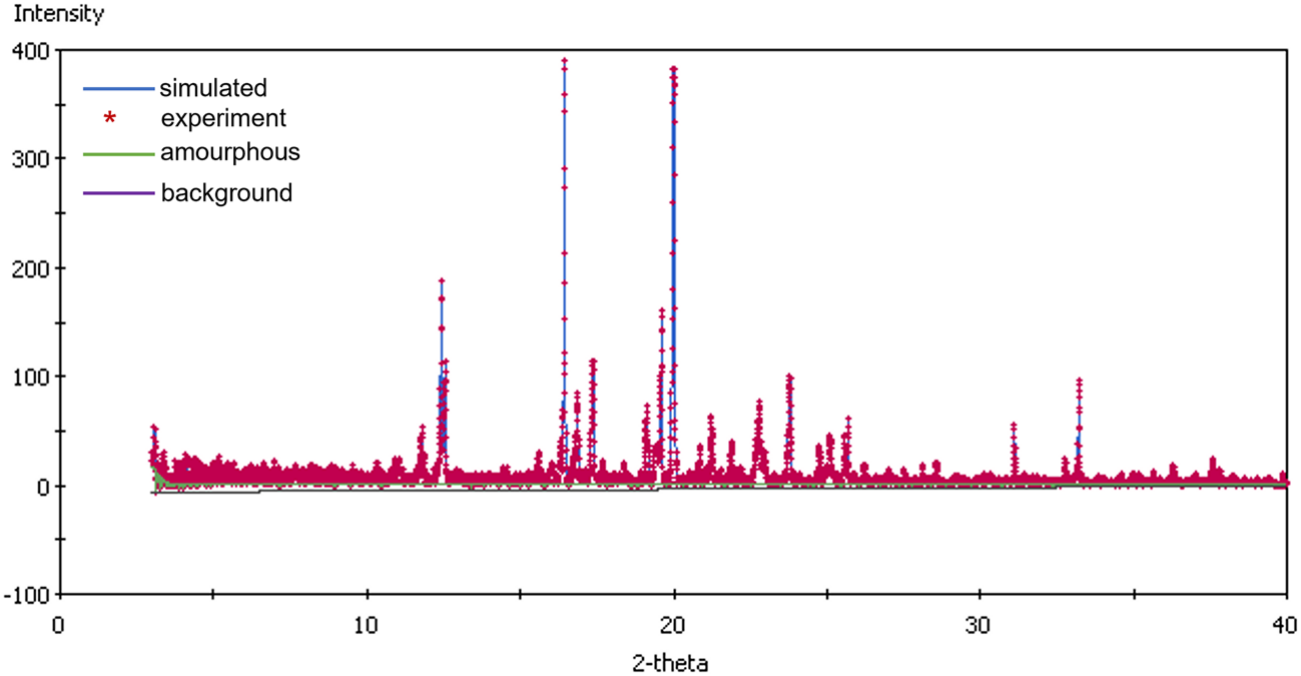
PXRD pattern decomposition and crystallinity degree (%)for KTZ-AA—Lactose sample after 3 months storage at 40°C and 75% RH (Degree of crystallinity = 70.13%, Coverage = 100%).

An example of powder X-ray diffraction pattern decomposition and crystallinity degree (%) determination is illustrated in Fig. 7 for the KTZ-AA – Lactose mixture sample after storage in the climate chamber under accelerated conditions for 3 months.

### Molecular Docking Study

**Fig. 8:**
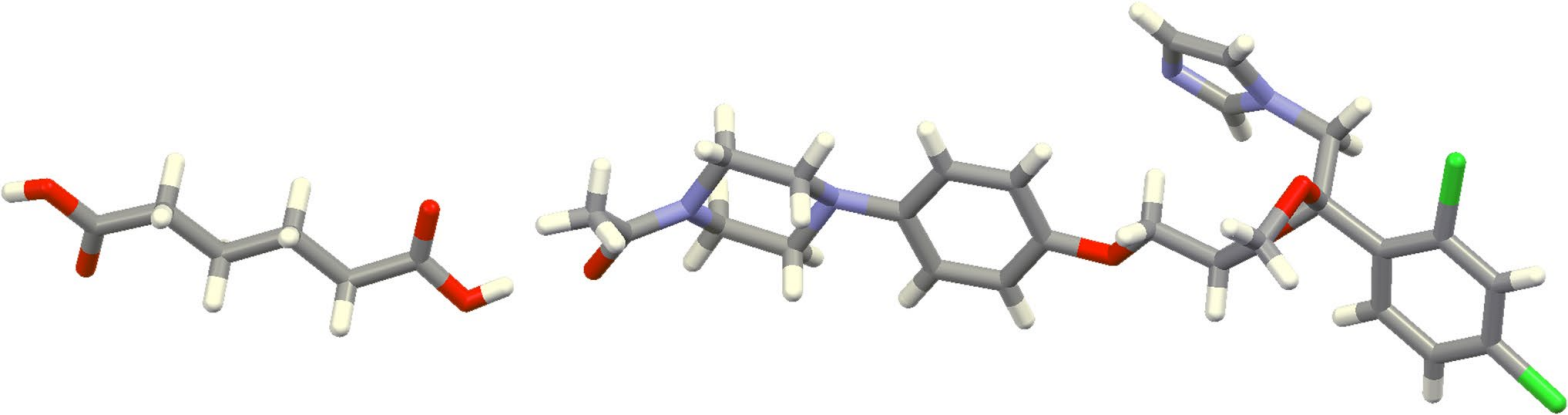
Optimized geometry of co-crystal model by Density Functional Theory calculations in gas phase at B3LYP/6-31G(d,p) level of theory.

The asymmetric unit of KTZ-AA co-crystal (YINWID code from Cambridge Structural Database) [38], pictured in Fig. 8, was selected as the representative model of the co-crystal for the molecular docking study.

Autodock Vina [59] is an automated tool designed to predict interactions between ligands and receptors. It systematically explores all degrees of freedom, particularly focusing on the torsion angles of the ligand, while employing a rapid grid-based energy evaluation. By integrating a search algorithm with an empirical free energy scoring function, the method narrows down numerous potential docked conformations to identify the most probable one, which is characterized by the lowest binding energy between the receptor and the ligand. Given the non-deterministic nature of the algorithm, each execution yields stochastic results. Consequently, for each ligand-receptor pair across the five systems (five ligands and one receptor), 10 binding modes were generated per execution. The exhaustiveness parameter was set at 12. The code was run 20 times, producing 200 docking conformations for each system. This methodology is consistent with our prior study [75], which aimed to characterize interactions between ligands and sterol 14α-demethylase as well.

The binding energies of KTZ, AA, KTZ-AA and posaconazole (the original ligand) to the sterol 14α-demethylase enzyme from *Candida albicans* (PDB id: 5FSA), as predicted through molecular docking, are listed in Table 5.

**Table V:** Binding Affinities of KTZ, AA, KTZ-AA Co-Crystal Model, and Posaconazole (Original Ligand) to 14-α Demethylase from *Candida albicans* (PDB id: 5FSA)

kcal mol^−1^	KTZ	AA	KTZ-AA	original
average E_bind_	−11.27	−5.15	−11.71	−12.07
SD	0.07	0.09	0.33	0.13
min	−11.40	−5.30	−12.10	−12.30
max	−11.20	−5.00	−11.00	−11.90

Binding affinities expressed as average values for the best conformer of the 20 code runs together with the standard deviation (SD), as well as the min and max values obtained within the 20 code runs.

*In silico* evaluations were performed on KTZ, AA, KTZ-AA co-crystal, and posaconazole to assess the influence of AA on KTZ’s affinity for sterol 14-α demethylase and its binding energy. The rationale behind including the co-former model despite the fact that the two molecules might dissociate in solution before reaching the binding site of the enzyme was that this theoretical model could be used as a template to design new solid-state structures. The results indicated that AA affects the binding energy of KTZ in the co-crystal form. Some of the conformations scored a binding energy of −12.10 kcal mol^−1^, but the average binding energy of the co-crystal is smaller by approximately 0.45 kcal mol^−1^ than KTZ. Despite the increase, the binding energy of KTZ-AA was almost 0.4 kcal mol^−1^ higher than that of posaconazole. The original ligand posaconazole scored a binding energy of −12.09 ± 0.13 kcal mol^−1^, indicating the highest affinity among the tested systems. Previous studies have also reported enhancements in KTZ affinity with *p*-hydroxybenzoic acid [76] and fumaric acid [75], although these enhancements were more substantial, of at least 1 kcal mol^−1^.

**Fig. 9:**
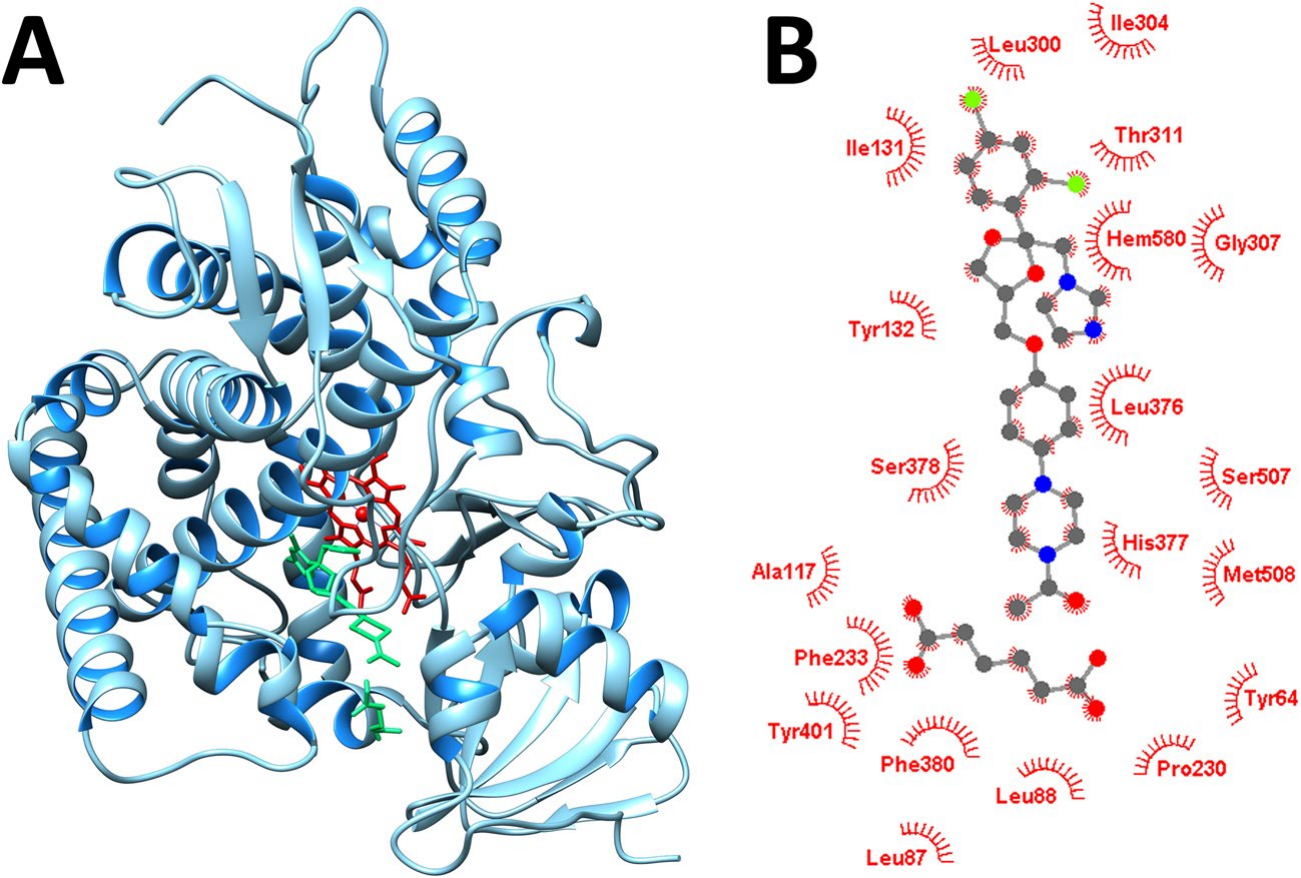
(**A**) Docked position of co-crystal KTZ-AA (green) to sterol 14α-demethylase from *Candida albicans* (PDB id:5FSA) together with (**B**) the 2D interaction diagram (ball-stick – KTZ-AA co-crystal) and residues involved in hydrophobic interactions.

As shown in Fig. 9A, the sterol 14α-demethylase binding site is located inside the macromolecule close to the heme group. Its pocket-like structure encourages ligands to bind in a geometrical specificity manner at the expense of other types of interactions. The amino acids PHE58, ALA61, ALA62, TYR64, GLY65, LEU88, TYR118, LEU121, TYR132, PHE126, ILE131, PHE228, PRO230, ILE231, PHE233, GLY303, GLY307, LEU376, HIS377, SER378, PHE380, TYR505, SER507, and MET508 define the site, as confirmed by the validation step.

Like fumaric acid, AA interacts with sterol 14α-demethylase by using its oxygen atoms to form hydrogen bonds with the TYR118, HIS377, and SER378 residues [75]. Additionally, the LEU121, PHE233, LEU376, ILE379, and MET508 residues create a number of hydrophobic interactions. KTZ forms a single hydrogen bond with HIS377 through its terminal oxygen atom. It also interacts hydrophobically with the residues TYR64, TYR118, PHE116, THR122, ILE131, TYR132, PHE228, PRO230, PHE233, GLY303, GLY307, HIS310, THR311, LEU376, SER378, PHE380, SER507, and MET508. The co-crystal interacts with the receptor only through hydrophobic interactions, with a number of additional residues, such as HIS377, TYR64, LEU87, LEU88, ALA117, LEU300, and TYR401, in addition to those mentioned above for KTZ.

### Reactivity Descriptors Based on DFT

The intermolecular interactions can be described by calculating the energies of the highest occupied molecular orbital (HOMO) and lowest unoccupied molecular orbital (LUMO). The HOMO energy reflects the electron-donating capability of a molecule, whereas the LUMO energy represents its electron-accepting potential. The HOMO and LUMO energies were calculated for KTZ, AA, KTZ-AA co-crystal, and the original ligand as key indicators of their chemical reactivity and stability, together with a series of quantum chemical reactivity descriptors, such as the ionization potential (I), electron affinity (A), HOMO–LUMO band gap (HLG), global hardness (η), global softness (σ) [77], electronegativity (χ) [78], chemical potential (μ), and global electrophilicity index (ω) [79, 80]. The results are listed in Table VI.

**Table VI:** Quantum Chemical Reactivity Descriptors Obtained on the Optimized Geometries of the Selected Ligands by Density Functional Theory Calculations in Gas Phase at B3LYP/6-31G(d,p) Level of Theory

eV	KTZ	AA	KTZ-AA	original
E_HOMO_	−5.073	−8.033	−5.173	−5.411
E_LUMO_	−0.998	−0.328	−1.020	−0.641
I	5.073	8.033	5.173	5.411
A	0.998	0.328	1.020	0.641
HLG	4.076	7.705	4.154	4.770
η	2.038	3.853	2.077	2.385
σ	0.491	0.260	0.481	0.419
χ	3.035	4.181	3.097	3.026
μ	−3.035	−4.181	−3.097	−3.026
ω	2.261	2.268	2.308	1.920

I–ionization potential; A–electron affinity; HLG–HOMO–LUMO gap; η–global hardness; σ–global softness; χ– electronegativity; μ–chemical potential; ω–global electrophilicity index.

HLG characterizes the charge transfer interactions between a molecule and its environment, with its value representing the required energy to remove an electron from the HOMO. A smaller HLG value indicates a faster reaction and serves as a key reactivity descriptor of molecular stability. Molecules with higher reactivity exhibit lower HLG values and increased softness values. The obtained HLG values presented in Table VI indicated that KTZ demonstrated greater reactivity than posaconazole, the original antifungal in complex with sterol 14α-demethylase. The reactivity of KTZ decreases slightly by 0.08 eV in its co-crystal form; this suggests that is marginally more stable than KTZ. However, overall, KTZ is more reactive than the original ligand posaconazole, both in pure and co-crystal forms. The HLG difference between KTZ or KTZ-AA and posaconazole was approximately 0.7 eV. Among the four compounds evaluated, AA was the most stable. Overall, the HLG values ≥ 3.54 eV indicate that all compounds are stable under normal conditions. The electrophilicity index, which quantitatively assesses a system’s ability to accept electrons, increased from posaconazole to co-crystals, indicating that posaconazole is less inclined to accept electrons, whereas co-crystals are more receptive to electron acceptance from their environment. Furthermore, the electron-accepting capacity of KTZ increased in its co-crystal form compared to its pure form, being more prone to accept electrons. An increasing of the electron-accepting capacity of co-crystal form of KTZ as compared to its pure form was also demonstrated in the case of ketoconazole-fumaric acid co-crystal [75].

## Conclusion

The compatibility study of the Ketoconazole-Adipic Acid co-crystal with the seven selected pharmaceutical excipients (magnesium stearate, lactose monohydrate, polyvinyl-pyrrolidone K90, microcrystalline cellulose, corn starch, colloidal silicon dioxide, and talc) was evaluated through various analytical techniques on 1:1 w/w physical binary mixtures.. The DSC results demonstrated compatibility between the co-crystal and six of the excipients employed. In the case of the co-crystal and MgSt mixture DSC revealed a change in the thermal behavior, suggesting the formation of a eutectic system. However, thermogravimetric analysis showed that the decomposition profile of the co-crystal remained unaffected in all binary mixtures. Complementary PXRD and FT-IR analyses also indicate no signs of chemical interactions between the co-crystal and the tested excipients under ambient conditions. Moreover, the KTZ-AA co-crystal maintained its chemical stability without degradation after three months storage under accelerated conditions (40°C/75% RH). Molecular docking demonstrated that co-crystallization of KTZ with AA modifies its binding affinity to sterol 14α-demethylase (CYP51) enzyme from the pathogenic yeast *Candida albicans*. KTZ-AA showed enhancing binding affinity compared to KTZ alone, though slightly less than posaconazole. Docking analysis revealed key hydrogen bonding and hydrophobic interactions contributing to ligand affinity. DFT-based reactivity descriptors indicated that KTZ is more reactive than posaconazole, both as single molecule or in co-crystal form. KTZ-AA exhibited increased electron-accepting capacity, suggesting altered reactivity upon crystallization. These findings support the potential of KTZ-AA co-crystal as improved antifungal candidate targeting CYP51.

The performed studies on the Ketoconazole-Adipic Acid co-crystal successfully demonstrated its potential as a solid oral dosage form, offering a promising alternative to the parent drug.

## Supplementary Information

Below is the link to the electronic supplementary material.

Supplementary file1 (DOCX 270 KB) 
