# Supplementary material for: Pharmaceutical Co-crystal of Ketoconazole-adipic Acid: Excipient Compatibility and In Silico Antifungal Potential Studies
Source: Pharm Res. 2025 Sep 4;42(9):1603–16. doi: 10.1007/s11095-025-03910-7 (PMC12507965; doi:10.1007/s11095-025-03910-7)
Supplement: Supplementary file 1 — Supplementary file1 (DOCX 270 KB) [file 11095_2025_3910_MOESM1_ESM.docx]

**Pharmaceutical co-crystal of Ketoconazole-Adipic Acid: excipient compatibility and in silico antifungal potential studies**

Flavia Martin^1^, Maria Miclaus^1^, Ana Maria Raluca Gherman^1^, Monica Dan^1^, Ioana Grosu^1^, Xenia Filip^1^, Irina Kacso^1a^

^1^ National Institute for Research and Development of Isotopic and Molecular Technologies, 67-103 Donat, 400293 Cluj-Napoca, Romania

^a^ Corresponding author: [irina.kacso@itim-cj.ro](mailto:irina.kacso@itim-cj.ro), +40264584037

**Table S1.** The characteristic absorption bands, in 1750-700 cm^-1^ spectral domain, of pure KTZ-AA and of the physical mixtures of KTZ-AA with excipients [67–70]

| **Vibrational bands positions (spectral domain 1750-700 cm^-1^)** | | | | | | | | **Assigment** |
| --- | --- | --- | --- | --- | --- | --- | --- | --- |
| **KTZ-AA** | **+MgSt** | **+Lactose** | **+PVP K90** | **+MCC** | **+Starch** | **+SiO_2_** | **+Talc** |  |
| 1708 | 1705 | 1708 | 1708 | 1707 | 1709 | 1708 | 1707 | C=O str. |
| 1618 | 1612sh | 1617 | 1617 | 1617 | 1618 | 1618 | 1617 | C=O str. |
| 1511 | 1511 | 1512 | 1512 | 1512 | 1512 | 1512 | 1512 | C=C aromatic group,  C-O stretch of cyclic ether |
| 1460 | 1464 | 1456 | 1456 | 1456 | 1458 | 1457 | 1457 | C=N arom. symm. stretch. |
| 1267 | 1266 | 1262 | 1262 | 1265 | 1264 | 1261sh | 1264 | C=C aromatic stretch,  COO- asymm stretch |
| 1243 | 1243 | 1243 | 1243 | 1242 | 1238 | 1239sh | 1239 | C=C-O stretch |
| 1183 | 1183 | 1183 | 1183 | 1183 | 1183 | 1184 | 1183 | C-O stretch of cyclic ether |
| 1105 | 1105 | 1111sh | 1111sh | 1105 | 1105 | 1106 | 1103 | C-N stretch |
| 1053 | 1052 | 1056 | 1056 | 1052 | 1050 | 1054 | 1048sh | C-O stretch of aliphatic ether |
| 997 | 996 | 993sh | 993sh | 998 | 999 | 999 |  | C-H stretch |
| 977 | 977 | 976sh | 976sh | 980 | 979sh | 978 | 977sh | C=C-H out of plane bending |
| 827 | 826 | 825 | 825 | 825 | 825 | 825 | 825 | C–Cl stretch |





**Figure S1.** PXRD patterns of the KTZ-AA co-crystal and binary mixtures of KTZ-AA with tested excipients

after 3 months storage at elevated temperature and humidity (40°C and 75% RH)
